# Design of A High Performance Zeolite/Polyimide Composite Separator for Lithium-Ion Batteries

**DOI:** 10.3390/polym12040764

**Published:** 2020-04-01

**Authors:** Yanling Li, Xiang Wang, Jianyu Liang, Kuan Wu, Long Xu, Jun Wang

**Affiliations:** 1 School of Materials Science and Engineering, Wuhan University of Technology, Wuhan 430070, China; liyanling@whut.edu.cn (Y.L.); wuwide@163.com (K.W.); dragonxu2012@163.com (L.X.); wgdfrp@whut.edu.cn (J.W.); 2 Department of Mechanical Engineering, Worcester Polytechnic Institute, 100 Institute Road, Worcester, MA 01609, USA

**Keywords:** zeolite, polyimide, phase inversion, spongy-like, lithium-ion batteries

## Abstract

A zeolite/polyimide composite separator with a spongy-like structure was prepared by phase inversion methods based on heat-resistant polyimide (PI) polymer matrix and ZSM-5 zeolite filler, with the aim to improve the thermal stability and electrochemical properties of corresponding batteries. The separator exhibits enhanced thermal stability and no shrinkage up to 180 °C. The introduction of a certain number of ZSM-5 zeolites endows the composite separator with enhanced wettability and electrolyte uptake, better facilitating the free transport of lithium-ion. Furthermore, the composite separator shows a high ionic conductivity of 1.04 mS cm^−1^ at 25 °C, and a high decomposition potential of 4.7 V. Compared with the PP separator and pristine PI separator, the ZSM-5/PI composite separator based LiFePO_4_/Li cells have better rate capability (133 mAh g^−1^ at 2 C) and cycle performance (145 mAh g^-1^ at 0.5 C after 50 cycles). These results demonstrate that the ZSM-5/PI composite separator is promising for high-performance and high-safety lithium-ion batteries.

## 1. Introduction

Lithium-ion batteries have been extensively applied in portable electronic devices because of several advantages, such as high energy density and long cycle life, along with low self-discharge rate [1,2,3]. In recent years, the applications field of lithium-ion batteries has been expanded to electric vehicles (EV), but this puts higher demands on the safety of lithium-ion batteries [4,5,6]. In lithium-ion batteries, the separator is an important part that serves as a physical barrier to separate the anode and cathode, as well as a channel for the transport of lithium-ions. Currently, polyolefin separators have been widely used in commercial lithium-ion batteries due to their good chemical stability, significant mechanical property, and excellent thermal shutdown function, as well as being cost-effective [7]. However, polyolefin separators generally lack the thermal stability due to their inherent low-melting-point characteristics, which may lead to safety problems, including fire and even an explosion. Furthermore, the inferior compatibility between nonpolar polyolefin separator and polar electrolytes results in poor electrolyte wettability, which increases the internal resistance and also restricts rate performance [8]. Therefore, developing an advanced separator with good wettability and improved thermal stability is greatly required for lithium-ion batteries.

In recent years, extensive efforts have been devoted to overcoming these shortcomings of polyolefin separators, most of which are based on the composite separator. Benefiting from the combination of outstanding characteristic of two or more materials, the composite separator usually exhibits excellent comprehensive properties. Typically, the types of composite separators include the combination of polymers with inorganic nanoparticle and the mixing between different polymers [9,10]. At present, in addition to advanced composite technologies, researchers have begun to focus on exploiting novel raw materials.

Zeolite, a kind of microporous aluminosilicate with a three-dimensional network structure, has been used as an inorganic component of separator due to its excellent electrolyte compatibility. Specifically, not only can zeolites interact with electrolytes for Lewis acid/base interactions, but their unique internal channels can also provide extra paths for lithium-ion transport, thereby increasing the charge–discharge properties of battery [11]. Zeolites are usually introduced by coating a polyolefin separator or a nonwoven mat. For example, Xiao et al. [12] coated polyethylene terephthalate (PET) nonwoven with NaA coating layers, and the thermal resistance and wettability were distinctly enhanced due to the presence of zeolite coating. However, the inorganic coating increases the thickness of the separator, which may reduce the capacity density of the battery, and inorganic nanoparticles easily to fall off, making them not conducive to cycle performance [13,14,15,16,17]. Some studies have directly embedded zeolites into polymer substrates to produce composite separators. Those separators are prepared by dispersing inorganic particles in a polymer solution in advance and followed by a forming process of the composite porous membrane. Zhang et al. [18] reported a zeolite/polyvinylidene fluoride (PVDF) composite separator. The result shows that the high electrolyte uptake and developed microporous structure of prepared separator yielded an improved rate capability and cycle performance. J. Nunes-Pereira et al. [19] prepared a NaY/polyvinyledenedifluoride-trifluoroethylene (PVDF-TrFE) separator with high porosity that exhibited superior ionic conductivity. However, the abovementioned composite separator prepared by semi-crystalline PVDF or its copolymer generally exhibited poor mechanical strength, which cannot guarantee the safety of the battery under harsh conditions. Polyimide (PI) is known to be an ideal material due to its excellent thermal stability and mechanical property. If PI is used as a polymer matrix, the mechanical strength of composite separator will be effectively improved.

On the basis of the above analysis, the zeolite/PI composite separator based on ZSM-5 zeolite and PI was prepared by phase inversion, to improve the comprehensive performance of the battery. The ZSM-5 zeolite acts as a filler to improve electrolyte wettability and electrolyte uptake. The PI serves as a skeletal support to provide the separator with high mechanical strength and thermal stability. The safety and electrochemical performance of the composite separator were evaluated by the characterizations of morphology, mechanical properties, thermal stability, and battery charge–discharge test, and they were compared with commercial polyolefin separators.

## 2. Materials and Methods 

### 2.1. Materials

First, 2,2-bis[4-(4-aminophenoxy)phenyl]propane (BAPP) and 4,4′-oxydianiline (ODA) were purchased from Aladdin Chemistry Co., Ltd. (Shanghai, China). Then, 4,4′-oxydiphthalic anhydride (ODPA) was purchased from Changzhou Yangguang Pharmaceutical Co., Ltd. (Changzhou, China). The ZSM-5, with an average size of 150 nm, was obtained from Nankai University Catalyst Co., Ltd. (Tianjin, China). *N*,*N*-dimethylacetamide (DMAc) was provided by Sinopharm Chemical Reagent Co., Ltd. (Beijing, China). A commercial PP separator (Celgard 2400, the thickness of 25 µm) was chosen for comparison.

### 2.2. Synthesis of ZSM-5/Polyamide Acid (PAA) Blend Precursors

ZSM-5/PAA blend precursors were prepared via in situ polymerization. Complete details of the preparation process are as follows. At first, a calculated amount of ZSM-5 was introduced to 10 mL of DMAc and stirred violently for 3 h, under ultrasonic, to form a ZSM-5 suspension. ODA and BAPP were then added to a three-necked flask containing different concentrations of the ZSM-5 suspension. Once ODA and BAPP were dissolved thoroughly, ODPA was added stepwise into the solution, and solid content of reaction solution was controlled to 12 wt% with extra DMAc. The reaction was carried out under nitrogen, to ensure a higher molecular weight. After continuously stirring for 8 h, we obtained a homogenous ZSM-5/PAA blend precursor solution.

### 2.3. Preparation of the ZSM-5/PI Composite Separator

The ZSM-5/PI composite separator was prepared from the ZSM-5/PAA blend solution via a phase inversion and thermal imidization. Specifically, the ZSM-5/PAA solution was cast on a glass substrate, using a casting knife with a gap of 100 μm. Subsequently, liquid film was exposed to a chamber, at a relative humidity (R.H.) of 80%, for 20 min, to induce phase separation and form a porous film, i.e., ZSM-5/PAA composite porous film. To extract the remaining DMAc, the porous film attached to the glass plate was delivered into an ethanol bath for 10 min, during which the film was automatically peeled from the glass plate. Prior to thermal imidization, the ZSM-5/PAA composite porous film was washed three times with water. After that, the film was treated at 80 °C for 1 h, 100 °C for 1 h, 200 °C for 1 h, and 280 °C for 1 h, respectively. As a result, a series of ZSM-5/PI composite separators with various content of ZSM-5 was obtained. For convenience, the resulting composite separators with the ZSM-5 content of 0 wt%, 5 wt%, 10 wt%, and 15 wt% were defined as PI, Z/PI-5, Z/PI-10, and Z/PI-15, respectively. The thickness of resultant separators varies from 27 to 32 μm, as shown in Table 1.

### 2.4. Characterization

Fourier transform infrared spectrum (FT-IR, Nexus670, Thermo Nicolet, Waltham, MA, USA) was applied to certify the structural transformation of PAA to PI. The tests were performed at a frequency range of 4000–400 cm^−1^. Scanning electron microscopy (SEM, JSM-IT300, JEOL, Tokyo, Japan) was employed to surveying microporous morphology on the surface and cross-section of the separators. The samples for morphology characterization were sputtered with a gold layer. Energy-dispersive spectroscopy (EDS) was used to further observe the element distribution of the separator.

Porosity is defined as the percentage of the total volume of space in the separator, which is usually determined by n-butanol adsorption. Since the character of n-butanol is similar to that of the electrolyte, the volume occupied by n-butanol after soaking is the porosity of the separator. The separator was soaked in n-butanol for 2 h and weighed before and after n-butanol absorption. The porosity of the separators was calculated by Equation (1):

Porosity(%) = (m_wet_ − m_dry_)/(ρ*V), (1)

where m_dry_ is the weight of the initial separator, m_wet_ is the weight of the separator after absorbing n-butanol, ρ is the density of n-butanol, and V is the volume of the initial separator.

The mechanical strength of separators was determined by a universal testing machine (Instron 5967, Instron Pty Ltd., Norwood, MA, USA), at a speed of 2 mm min^−1^. The samples with the dimensions 50 mm × 10 mm were used. The effective length of the samples was 20 mm.

The thermal stability of the separators was studied by thermal gravimetric analyzer–differential scanning calorimeter (TGA–DSC, STA 449F3, Netzsch, Selb, Germany) over the temperature range of 50–800 °C, at a heating rate of 10 °C min^−1^. For the thermal shrinkage measurement, the separator was heated in an oven, at 25 °C, 130 °C, 150 °C, and 180 °C, for 30 min, and the shape change was surveyed after being heated.

The contact angle meter (OCA20, Dataphysics, Filderstadt, Germany) equipped with a video capture was employed to measuring the contact angle of separator toward electrolyte. The electrolyte uptake of the separators was determined by measuring the weight change before and after being soaked in the electrolyte (1 M LiPF_6_/EC:DMC (1/1, *v*/*v*)) for 2 h, and then calculated by Equation (2):

Electrolyte uptake(%) = (W − W_0_)/W_0_×100% (2)

where W_0_ and W are the weights of the separators before and after being soaked in an electrolyte, respectively.

The electrochemical stability and ionic conductivity were examined on an electrochemical analyzer (Ivium Stats, Ivium Technologies, Eindhoven, Netherlands). For electrochemical stability measurement, the separator was sandwiched between lithium metal and stainless-steel electrodes and then assembled into a CR2032 coin cell. The assembled cells were submitted to linear sweep voltammetry (LSV) measurement to obtain electrochemical stability windows of the separators. The measurements were carried at the scan rate of 5 mV s^−1^ over the voltage range of 2.5–5.5 V. The ionic conductivity of the separators was determined by electrochemical impedance spectroscopy (EIS), with two stainless-steel blocking electrodes. The AC impedance spectra were measured in the frequency range from 0.1 to 10^6^ Hz. The ionic conductivity was calculated by Equation (3):

σ = d/(R*S)(3)

where d is the average thickness obtained by measuring three different regions of the separators. R represents the bulk resistance, which is determined by the high-frequency intercept on the real axis in the AC impedance spectrum. S is the effective area of the separators, that is, the area of the stainless steel.

Cycle and rate performances were measured by using CR2032 coin cells, where the lithium metal was used as an anode, and the LiFePO_4_ was used as a cathode. The measurements were performed by battery-testing equipment (CT2001A, LAND Electronics, Wuhan, China). The cycle test was performed at a charge/discharge rate of 0.5 C/0.5 C for 50 cycles. Rate capability test was conducted on the discharge of 0.2, 0.5, 1, 2, and 0.2 C, under 2.5–4.2 V.

## 3. Results

**Figure 1:**
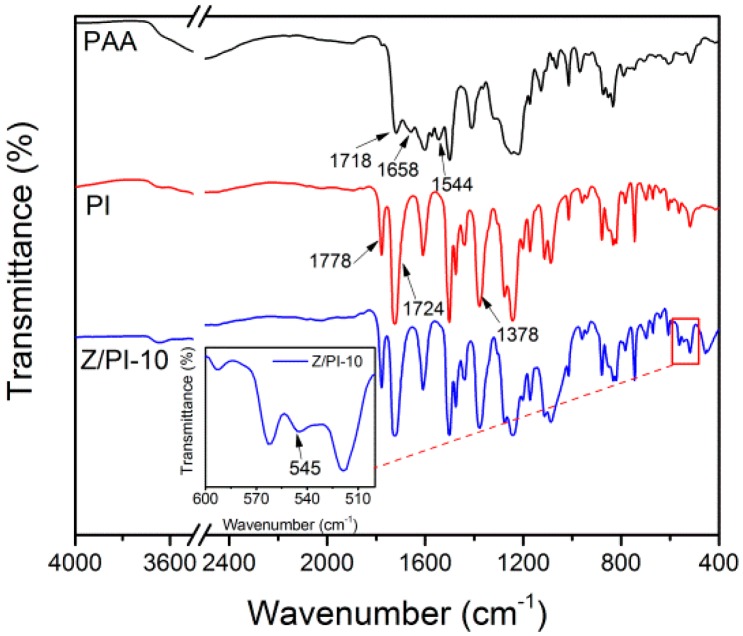
FT-IR spectra of PAA, PI, and Z/PI-10 separators.

FT-IR spectra of PAA, PI and Z/PI-10 separators were carried out to confirm the success of complete imidization of PI and smooth incorporation of ZSM-5 zeolite, as shown in Figure 1. In the infrared spectrum curve of PAA, the peak at 1718 cm^−1^ can be assigned to the C=O in COOH groups. The peaks at 1658 and 1544 cm^−1^ are attributed to stretching vibrations of C–NH and C=O in CONH, respectively. After heat treatment, new peaks appeared at 1778, 1724, and 1378 cm^−1^, assigned to C=O asymmetric stretching, C=O symmetric stretching, and C–N stretching of PI, respectively [20]. At the same time, the characteristic bands of PAA at 1718, 1658, and 1544 cm^−1^ were not found, indicating that the PAA was fully transferred to PI after the imidization. Besides, a five-membered ring characteristic absorption peak of ZSM-5 appeared at 545 cm^−1^, indicating that ZSM-5 is successfully introduced into the PI bulk.

**Figure 2:**
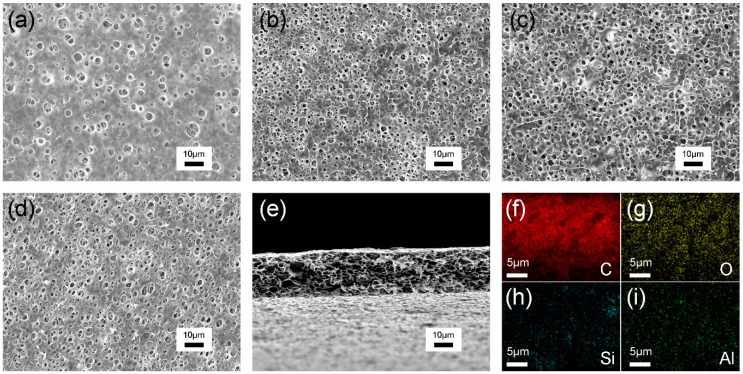
SEM micrographs for the surface of PI separators and composite separator with different ZSM-5 concentrations: (**a**) PI, (**b**) Z/PI-5, (**c**) Z/PI-10, and (**d**) Z/PI-15. (**e**) SEM micrographs for the cross-section of Z/PI-10 composite separator. (**f**–**i**) Elemental mapping of Z/PI-10 composite separator.

Figure 2 presents morphologies of the pristine PI separator and composite separator obtained by coagulating at a condition of 80% humidity. As can be seen from Figure 2a–d, the incorporation of ZSM-5 with different contents has a slight effect on the pore morphologies. In Figure 2a, the PI separator without ZSM-5 has few pores and non-uniform pore size distribution, which could not provide a sufficient path for ions transport [21,22]. After adding a certain amount of ZSM-5 into the PI separator, the surface morphologies of the separators are changed due to the internal cavities of ZSM-5 nanoparticles and the micro-voids formed between the nanoparticles. When adding 5% and 10% ZSM-5, the size of the pores becomes smaller and the connectivity among the pores gets better, which will facilitate ions’ migration and hinder the development of lithium dendrites [23], as displayed in Figure 2b,c. With the content of ZSM-5 further increasing to 15% (Figure 2d), the composite separator exhibits an increased pore size with an almost unchanged number of pores, which may lead to a decrease in tensile strength that is not robust enough for the practical application. In the cross-sectional view of Z/PI-10 composite separator (Figure 2e), a greatly interconnected spongy-like pore structure is observed, which originates from the relatively slow phase separation process of the polymer/solvent system [21,24]. Figure 2f–i gives the typical element mapping of section–section of Z/PI-10 composite separator. It is clear that C, O, Si, and Al elements are uniformly dispersed in the separator, indicating that zeolites are evenly introduced to the separator.

**Figure 3:**
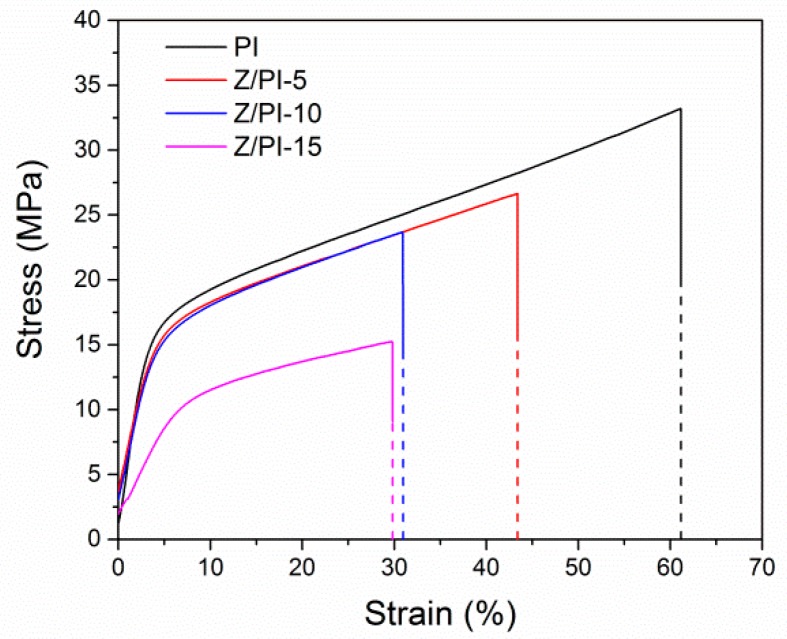
The stress–strain curves of different separators.

To further characterize the microporous structure of the separators, the porosity of the PP separator, PI separator, and composite separator were studied by the n-butanol absorption method, and the results are summarized in Table 1. It can be found that ZSM-5 has a positive effect on the porosity of separators. The porosity of the pristine PI separator (45%) was higher than that of PP separator (41%). More specially, the porosity increased to 53%, 59%, and 61% for the Z/PI-5, Z/PI-10, and Z/PI-15 composite separator as we increased the corresponding content of ZSM-5 nanoparticles in separators. The improved porosity can be ascribed to the internal cavities of ZSM-5 nanoparticles and the porous structure of the polymer matrix affected by the incorporation of ZSM-5. Unlike the porosity, the tensile strength of the separators decreased gradually with the increase of ZSM-5. As depicted in Figure 3, the mechanical strengths dropped from 33 MPa of the pristine PI separator to 15 MPa of the Z/PI-15 composite separator. The sacrificed tensile strengths can be explained by the improved porosity, which reduced the connection points among the three-dimensional network [22]. The desirable tensile strength of the separators should be higher than 7 MPa [17]; thus, the prepared separators are qualified for practical application in lithium-ion batteries. Considering the satisfactory porosity, which contributes to the electrochemical performance, and credible mechanical strength, which guarantees the safety of battery during the assembly and operation, the Z/PI-10 separator was selected as a representative sample of the composite separator for subsequent studies.

**Figure 4:**
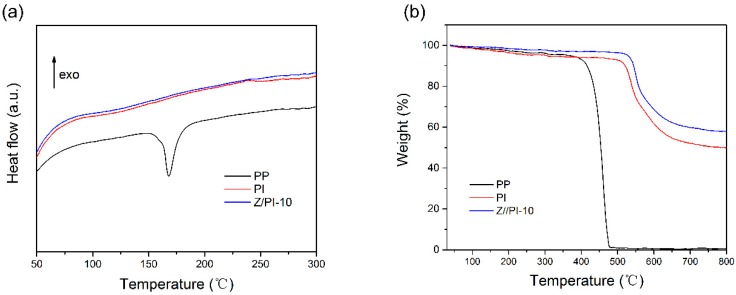
(**a**) DSC and (**b**) TGA curves of PP, PI, and Z/PI-10 separator.

The thermal stability of the separators is an important indicator to assess the safety of the battery. DSC was carried out to evaluate the thermal behaviors of the prepared separators. From the DSC profile of the PP separator, shown in Figure 4a, an obvious endothermic peak at about 167 °C is detected, which is the melting temperature of PP [25,26]. In contrast, no obvious peaks are observed in the range of 50–300 °C for both the pristine PI separator and Z/PI-10 composite separator, and there is a negligible difference among the PI separator and Z/PI-10 composite separator, indicating that the PI-based separators possess significant thermal stability up to 300 °C. Meanwhile, to further evaluate the thermal behaviors of the separators, a TGA test was performed, and the results are shown in Figure 4b. It can be found that the PP separator develops a sharp degradation at around 400 °C, and it thoroughly decomposes at above 475 °C. However, the weight loss of the pristine PI separator is only 7.1% before 500 °C, which is caused by the evaporation of moisture uptake. Meanwhile, the presence of ZSM-5 can further enhance the resistance to heat of composite separator [27]. As a result, the Z/PI-10 composite separator also exhibits improved thermal performance with a weight loss of 3.5% before 500 °C. From the results of the DSC and TGA tests, it can be confirmed that the PI separator and Z/PI-10 composite separators have excellent heat resistance.

**Figure 5:**
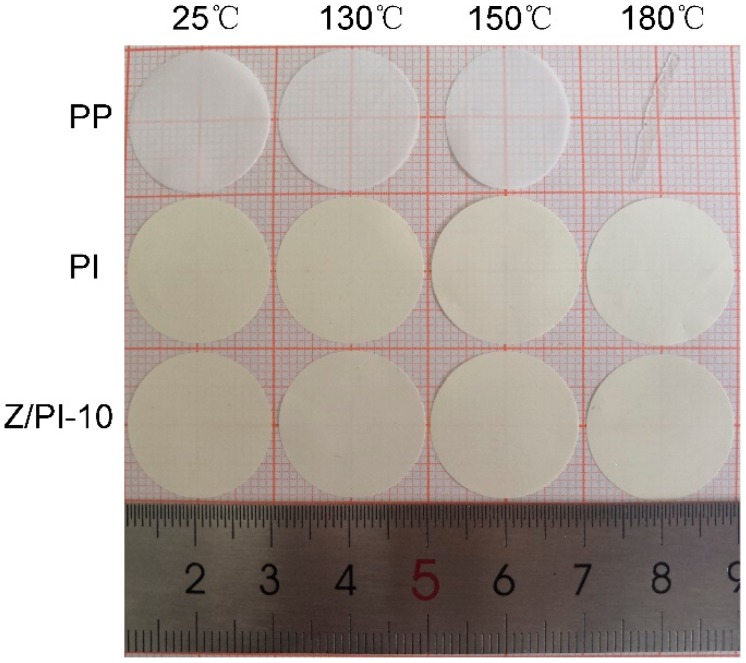
Thermal shrinkage behaviors of the separators after heat treatment at different temperatures for 30 min.

The separators should maintain shape integrity to prevent contact between the two electrodes at elevated temperature; otherwise, it may trigger a short circuit and even an explosion [28,29]. To investigate the thermal shrinkage of the selected separators, heat treatment test was adopted. Specifically, the PP, PI, and Z/PI-10 separators were heated at different temperatures, ranging from 25 to 180 °C, for 30 min. Figure 5 reveals the evolution of dimensional with various temperatures. The PP separator begins to shrinkage at 130 °C due to its internal stress caused by uniaxial stretching. When the temperature reaches 180 °C, the PP separator experiences a more severe shrinkage and its color changes to transparent. In contrast, the overall shapes of PI separator and Z/PI-10 composite separator remain unchanged after being treated at 180 °C, which is mainly due to the stable aromatic heterocyclic structure existing in PI molecular chains. Obviously, both the PI separator and Z/PI-10 composite separator are expected to improve the safety of the corresponding battery.

**Figure 6:**
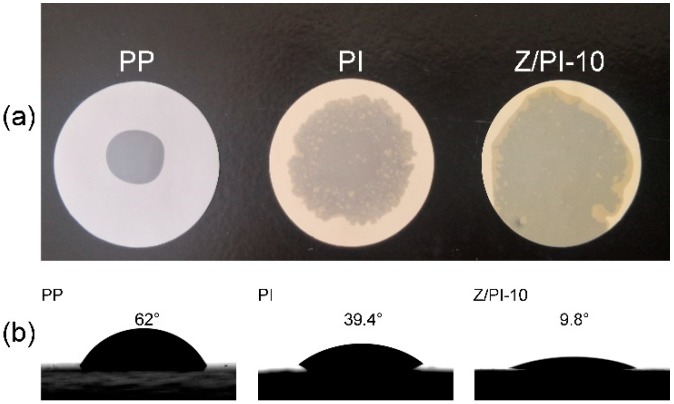
(**a**) Photograph showing the wetting behavior of the separators wetted by liquid electrolyte. (**b**) Contact angle test of PP, PI, and Z/PI-10 separators with liquid electrolyte.

A separator with high wettability is critical for a battery, as it can provide a channel for ions’ migration by absorbing and holding sufficient electrolytes [30]. The electrolyte wettability was illustrated by observing the diffusion area of the electrolyte on the separator. As shown in Figure 6a, most areas of the Z/PI-10 composite separator were infiltrated by the electrolyte, while the PP separator was hardly wetted and the electrolyte generated a droplet on its surface. The wettability of different separators was further studied by the contact angle concerning the electrolyte (Figure 6b). Usually, the smaller contact angle suggests the better affinity with liquid electrolyte [31]. As can be seen, the contact angle of the Z/PI-10 composite separator is 9.8°, which is much lower than the values of PI separator (39.4°) and PP separator (62°), implying that the Z/PI-10 composite separator possesses the best electrolyte wettability. This result can be attributed to the high porosity produced by highly interconnected spongy-like pore structure and excellent electrolyte affinity provided by ZSM-5, helping absorb more liquid electrolyte [32,33]. This superior wettability helps to improve ions’ transport in the electrolyte, and thus enhancing the electrochemical performance of the corresponding battery.

**Figure 7:**
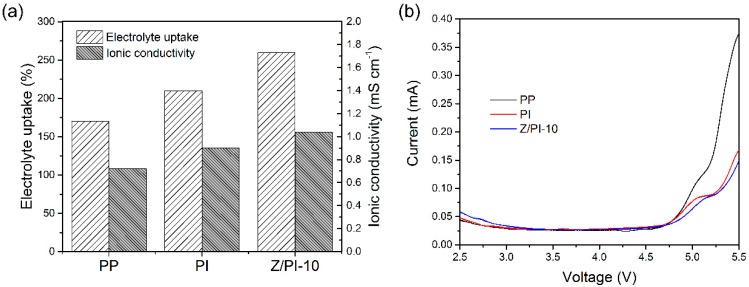
(**a**) Electrolyte uptake and ionic conductivities of different separators. (**b**) Linear scan voltammograms (LSV) curves of different separators.

The electrolyte uptake of the obtained separators is presented in Figure 7a. The Z/PI-10 composite separator showed a higher electrolyte uptake of 260% than that of PI separator (210%) and that of PP separator (170%). These results are consistent with the results of the contact angle test. In general, the higher electrolyte uptake leads to higher ionic conductivity, which has been reported in previous literature [26,28,30]. As shown in Figure 7a, the ionic conductivities are considered to be 0.72 mS cm^−1^ for PP separator, 0.9 mS cm^−1^ for PI separator, and 1.04 mS cm^−1^ for Z/PI-10 separator, which has the similar trends as the electrolyte uptake. It can be seen that the incorporation of ZSM-5 improves the conductivity of the separator, which is attributed to abundant interconnected micropores of ZSM-5 nanoparticles providing more room for the storage of liquid electrolytes, as well as Lewis acid–base interaction between ZSM-5 and electrolyte facilitating the transport of lithium-ion. High ionic conductivity usually represents low internal resistance, thereby helping to improve electrochemical behaviors.

The electrochemical property of the separators was tested by LSV methods, as presented in Figure 7b. Here, the electrochemical stability of the separators was determined by the voltage corresponding to the abrupt increase in current caused by the redox reactions [34]. For all the separators, no decomposition is observed below 4.7 V, suggesting that the pristine PI separator and Z/PI-10 composite separator are stable up to 4.7 V, which is related to the good affinity of separator toward electrolyte. This extensive electrochemical window makes it possible to be used in high-power batteries.

**Figure 8:**
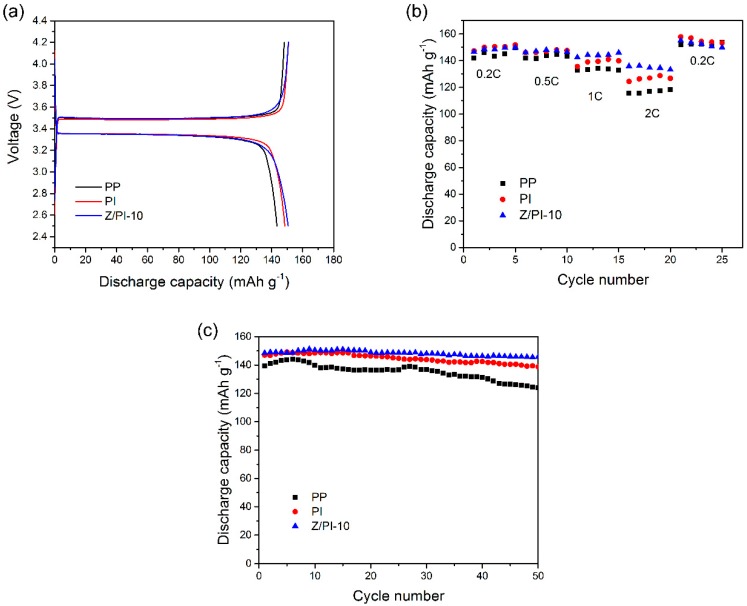
The electrochemical behaviors of LiFePO_4_/Li cells made with PP separator, pristine PI separator, and Z/PI-10 composite separator. (**a**) Initial charge/discharge (0.2 C/0.2 C). (**b**) Rate performances (0.2 C/0.2–2 C). (**c**) Cycle performances (0.5 C/0.5 C).

Figure 8a compares the initial charge–discharge profiles of the cells assembled with PP separator, PI separator, and Z/PI-10 composite separator with voltage ranging from 2.5 to 4.2 V, at 0.2 C. It is noticed that all the separators have relatively flat and long charge–discharge platforms, indicating that the cells assembled by those separators can maintain stable voltage and low polarization in the time of charge and discharge [32]. More importantly, the cells assembled with PI separator (148 mAh g^−1^) and Z/PI-10 composite separator (151 mAh g^−1^) have a slightly higher initial discharge capacity than PP separator (143 mAh g^−1^) at the first cycle.

Regarding rate capabilities tests, the cells with PP separator, pristine PI separator, and Z/PI-10 composite separator were discharged at current densities ranging from 0.2 to 2 C, and the results are depicted in Figure 8b. For all the separators, the discharge capacities tend to decrease with increasing current density, which is caused by the polarization [35]. Meanwhile, the cells with Z/PI-10 composite separator always represent higher discharge capacities compared to those with PI separator and PP separator at the same C rates, and the gaps in discharge capacities among the three separators become more pronounced as the current density increases from 0.2 to 2 C. In detail, when the current reaches to 2 C, the PP separator shows an inferior capacity of 117 mAh g^−1^, while the PI separator and Z/PI-10 composite separator maintain the capacity of 127 and 133 mAh g^−1^, respectively. Interestingly, when the density returns to 0.2 C, the discharge specific capacity of all the separators recovers to its original capacity of 0.2 C. This excellent discharge capacity of cells with the Z/PI-10 composite separator may be related to superior ionic conductivity, which contributes to the rapid diffusion of ions in the battery [36].

For the cycling tests, the cells with three different separators were conducted at a 0.5 C rate, as displayed in Figure 8c. At the first cycle, the discharge capacities of PP, PI, and Z/PI-10 separators were 139.5, 147, and 148.5 mAh g^−1^, respectively. With increasing cycle number, the discharge capacity of these cells declined gradually. After 50 cycles, the cells with PP separator deteriorated rapidly to 125 mAh g^−1^, with 89.6% capacity retention, and the cells with PI separator exhibited greater than 93% capacity retention. The cells employing Z/PI-10 composite separator still maintained a discharge capacity of 145 mAh g^−1^, accompanied with a capacity retention of 97.6%. This result indicates that the Z/PI-10 composite separator possesses the brightest cycle performance because it has extensive porosity and superior electrolyte retention, resulting in a reversible charge–discharge reaction.

## 4. Conclusions

In summary, we have successfully prepared a ZSM-5/PI composite separator via a phase inversion process. The uniform spongy-like porous separator was obtained as a result of coagulating at a condition of 80% humidity. The composite separator not only presents robust mechanical strength but also high porosity. In addition, due to the synergistic thermal effect of polyimide matrix and ZSM-5 zeolite, the Z/PI-10 composite separator exhibits satisfactory thermal durability, which is beneficial to improve the safety of batteries. Benefiting from better electrolyte wettability and electrolyte uptake, the Z/PI-10 composite separator shows high ionic conductivity compared to the pristine PI separator. The introduction of zeolites had no negative effect on the electrochemical stability of the separator, and the Z/PI-10 composite separator was stable above 4.7 V. More importantly, the cells based on the Z/PI-10 composite separator showed excellent electrochemical properties in both cycle performances and rate capabilities. This study suggests that ZSM-5/PI composite separator is a promising material for high-performance lithium-ion batteries, such as batteries for EV application.

## Figures and Tables

**Table 1:** The composition, thickness, and porosity of different separators.

Sample	ZSM-5/PI (*w*/*w*)	Thickness (μm)	Porosity (%)
PI	0	29	45
Z/PI-5	5/95	27	53
Z/PI-10	10/90	30	59
Z/PI-15	15/85	30	61
